# A technique to reduce skin toxicity in radiotherapy treatment planning for esophageal cancer

**DOI:** 10.1002/acm2.12812

**Published:** 2020-01-11

**Authors:** Wanfu Yang, Zhihua Yang, Ting Zhao, Wei Ding, Wei Kong, Pan Wang, Hongqiang Ye, Zixin Zhang, Jun Shang

**Affiliations:** ^1^ Department of Radiation Oncology General Hospital of Ningxia Medical University Yinchuan Ningxia China

**Keywords:** esophageal carcinoma, intensity modulated radiotherapy, skin toxicity, treatment planning

## Abstract

**Purpose:**

To demonstrate a specific skin dose limiting technique in radiotherapy treatment planning for esophageal cancer and carry out a comparative analysis combining with clinical cases.

**Material and methods:**

Thirty patients with cervical and upper thoracic esophageal carcinoma previously treated in our institution were selected. A treatment plan had been finished previously according to the planning parameters directives from physician and delivered for each patient. In this study, we copied the previously delivered plans in radiotherapy treatment planning system and converted a low dose level (usually 5Gy) to a skin dose limiting structure (SDLS), then we set the objective functions of the SDLS in the Pinnacle Inverse Planning module and re‐optimize the plans to reduce the skin doses. Finally, we compared the dose distribution and other parameters of target volume and organs at risk (OARs) between the old plans and the new plans.

**Results:**

There was no significant difference in most of OARs sparing. However, for all plans, the maximum dose to the SDLS decreased from 6145.90 ± 416.96 cGy to 5562.09 ± 616.69 cGy with maximum difference of 1361.30 cGy (*P* < 0.05), the percentage volume of 40Gy received by the SDLS decreased from (10.20 ± 6.36)% to (5.46 ± 4084)% with maximum difference of 9.89% (*P* < 0.05). For the target volume, there was no significant difference in the average dose and maximum dose, the approximate minimum dose to the target volume decreased from 5711.28 ± 164.61 cGy to 5584.93 ± 157.70 cGy (*P* < 0.05), the conformal index and homogeneity index of the target volume were hardly changed.

**Conclusion:**

In radiotherapy treatment planning for esophageal cancer patients, the skin dose can be significantly reduced using the skin dose limiting technique, and the impact on the dose to target volume and OARs is little, this technique can be used in most radiotherapy treatment planning.

## 1 INTRODUCTION

Esophageal cancer is one of the most common malignant tumors in the digestive tract. According to the latest global cancer report released by the World Health Organization in 2018, the incidence of esophageal cancer ranks seventh among all cancers (3.2 percent of new cancers in the world) and the mortality rate ranks sixth among all cancer deaths (5.3 percent of total cancer deaths).^1^ The main treatments for esophageal cancer include surgery, radiotherapy, and chemotherapy, and most of the esophageal cancer patients need radiotherapy throughout the course of the disease. With the emergence and fast development of intensity modulated radiotherapy, the planning and delivery of radiation techniques have been greatly improved. We now can get higher prescription dose and better dose conformity to the target volume, and the 5‐year survival rate of patients with esophageal cancer has been greatly improved.^2^, ^3^, ^4^, ^5^, ^6^ However, because the anatomic position of the target volume is close to the skin (especially for the cervical and upper thoracic esophageal carcinoma), skin toxicity is inevitable during the process of radiotherapy, the skin injury can negatively affect the quality of life of the patients.^7^, ^8^, ^9^ This skin reaction usually begins with the dose of 20–25 Gy, and radiation dermatitis occurs significantly after the cumulative dose to the skin reaches 40 Gy in the middle and late stages of radiotherapy.^10^ The mild symptoms include local erythema, dryness, and desquamation, and the severe symptoms will be local skin pain, edema and exudates, moist desquamation and so on.^11^, ^12^, ^13^, ^14^, ^15^ Radiation‐induced skin reactions occur as a result of damage to the basal cell layer of the skin and resulting in an imbalance between the normal production of cells in this layer and the destruction of cells at the skin surface.^16^, ^17^, ^18^ Although skin toxicity is inevitable in the process of radiotherapy, the dose to skin can be reduced as much as possible through ideal treatment planning, so as to reduce the degree of skin injury during radiotherapy. Within the last few years, multi‐criteria optimization, knowledge‐based planning approach (including model based planning, atlas‐based planning, dose‐volume histogram guidance planning and so on) have been used in Auto Planning, which is expected to improve the efficiency and quality of radiotherapy treatment planning.^19^, ^20^, ^21^, ^22^, ^23^ Although significant progress has been made in this area, much work is still needed to explore practical issues related to clinical implementation. For example, regions of tissue outside of delineated regions of interests may not be taken into account in auto planning, while a human planner may also optimize to reduce the skin dose. From the point of treatment planning method, this paper demonstrates a skin dose limiting technique in treatment planning for esophageal cancer patients.

## 2 MATERIAL AND METHODS

### 2.1 Patient selection and target volume contouring

A total of 30 patients with cervical and upper thoracic esophageal cancer treated in our hospital during January to December in 2018 were selected, among which 18 were male and 12 were female patients. The median age was 53 years and the Karnofsky Performance Status (KPS) score was 80 or more. They all showed severe skin reaction during the whole process of radiation treatment. All of these patients were simulated and immobilized with a thermoplastic mask, lying on the couch, placing hands across their elbows on the forehead. Computed tomography slices (5mm) were obtained from a large aperture CT scanner (Siemens SOMATOM Sensation Open; without Contrast) in the free and calm breathing state of the patients, the scanning range is from the skull base to the lower margin of the liver. When the scan was complete, the slices were sent to the Pinnacle treatment planning system v. 9.8(Philips Medical System, Milpitas, CA, USA). All the delineations of target volumes and organs at risk (OARs) were finished by an experienced physician and examined and approved by at least one senior physician. The length of target volume was 16–25 cm (mean 20. 68 ± 4.46 cm), and all of the patients were prescribed the same prescription dose 60 Gy at 2Gy/fraction to Planning target volume (PTV).

### 2.2 Treatment planning and utilization of skin dose limiting technique

After completing the contouring of target volume and OARs, a planning directive was completed. The planning directive outlined the physician’s planning guidelines including target prescriptions (60Gy/2Gy/30Fx, V_60Gy_ ≥ 95%, D_max_ < 66 Gy), normal structure goals (shown in Table 1), and other plan parameters. In clinical practice, we require that the dose should not be higher than 66 Gy. If it is inevitable, the volume of the dose above 66 Gy should not exceed 5% of the volume of PTV, and also should not be in the esophagus and trachea. Then the dosimetrists would complete a practicable treatment plan in accordance with the planning directives.

**Table 1:** Plan constraints of OARs in treatment planning for esophageal cancer.

	Spinal Cord	Lung Left/Right	Heart	Heart
Dose( Gy)	45	20	40	30
Percent volume (<=%)	0	25	30	40
D_mean_(Gy)		15		

OARs, Organs at risks.

**Figure 1:**
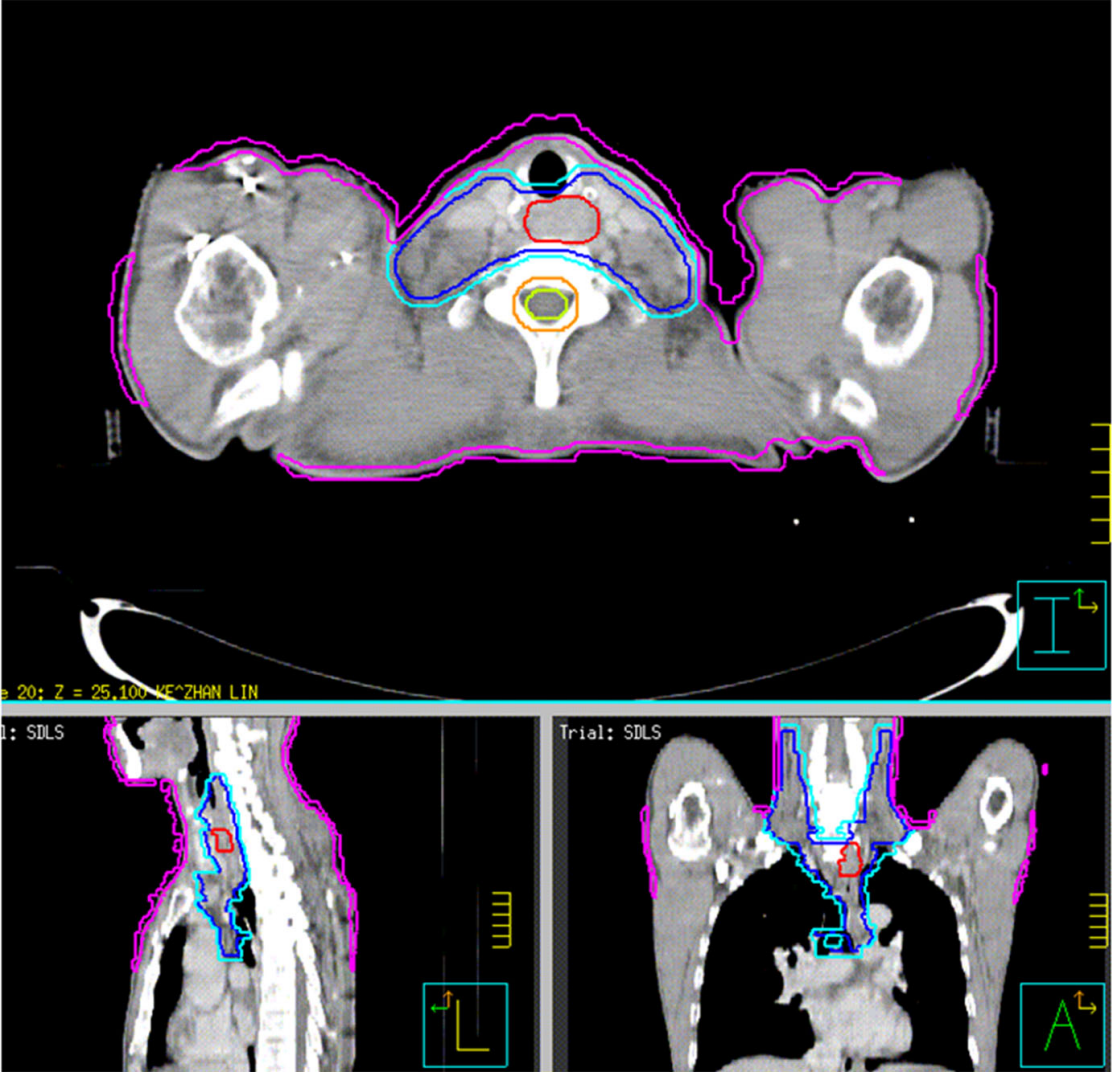
Skin dose limiting structure (Shown in purple).

The patients were planned with five fixed fields (or with seven fixed fields for those whose planning directives were difficult to meet), the gantry angle of fields were set to be 200°, 330°, 0°, 40°, and 160°for five fields or 200°, 260°, 310°, 0°, 50°, 100°, and 160°for seven fields. The photon energy was 6 MV, the machine was Elekta Precise. In the previously finished and delivered plans, the optimization objectives related to the skin dose include some manually created rings around the PTV to compress the isodose curves, but it made little contribution to reducing the skin dose. In this study, the already finished plans of the 30 patients were copied, and an isodose level of 5 Gy was generated and converted into a structure for each plan. The structure “Outline” (external contour of the patient) was then contracted 0.5cm to generate a structure “Outline‐0.5,” and then the structure generated by the 5 Gy isodose level subtracts the “Outline‐0.5” and any overlapping parts with the structure PTV, a skin dose limiting structure (SDLS) with a thickness of 0.5cm just inside the external contour of the patient was created (Fig. 1). Sometimes the SDLS can be manually modified in order to make it more practical.

After the SDLS had been created, it was added to the optimization objectives of the newly copied plan and the objective functions were set. In this study, the objective functions of SDLS were set as follows: D_max_ < 50 Gy, weight 20, V_40Gy_ < 5%, weight 30. After the new objective functions were set, the newly copied plan was re‐optimized and calculated to get a new dose distribution. In order to obtain a more ideal skin dose distribution, the optimization functions of the SDLS can be adjusted properly in different plans before re‐optimization on the premise that the dose distribution of target volume is not adversely impacted and the dose to OARs is not increased significantly.

### 2.3 Plan evaluation

**Figure 2:**
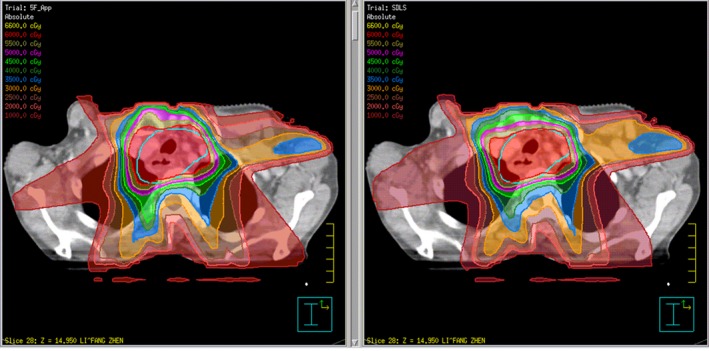
Old plan with dose colorwash (left) and the new plan with dose colorwash (right).

**Figure 3:**
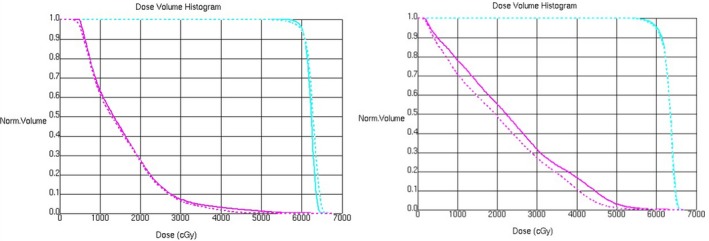
DVHs of both trials for worst (left) and best cases (right). The solid lines represent the old plan and the dashed lines represent the new plan, PTVs are shown in sky blue and the SDLSs are shown in purple. DVHs, dose‐volume histogram; PTVs. Planning target volumes; SDLSs, skin dose limiting structure.

After the full optimization and calculation of the newly copied plan had been completed, we reviewed the two plans (the old plan and the new plan) side by side in the Pinnacle Plan Evaluation module, paying close attention to the changes of dose to target volumes, OARs and normal tissues (Fig. 2). In order to get a quantitative analytical result, we reviewed the dose‐volume histogram (DVH) to determine the approximate maximum dose D_2%_, approximate minimum dose D_98%_, average dose D_mean_, conformal index (CI=(Vt,ref∗Vt,ref)/(Vref∗Vt), where Vt refers to the volume of PTV, Vt,ref refers to the volume of PTV covered by the isodose line of 60Gy, Vref refers to the volume covered by 60Gy isodose line) and homogeneity index ($HI=\left(D_{2\%}-D_{98\%}\right)\;/\;D_{50\%}$, where $\;D_{50\%}$ is the median absorbed dose of the PTV, $D_{2\%}$, and $D_{98\%}$ represent the dose received by 2% and 98% of the volume of PTV) of the PTVs of the two plans (Figure 3 shows the DVHs of both trials for the best and worst cases).^24^, ^25^, ^26^ Meanwhile, we compared the maximum dose and V_40Gy_ of the SDLS, the maximum dose of spinal cord, the D_mean_, V_5Gy_, V_10Gy_, V_20Gy_ of both lungs, and the D_mean_, V_30Gy_, V_40Gy_ of heart of the two plans. Patient‐specific quality assurance (QA) for all the treatment plans were performed using Mapcheck 2 (Sun Nuclear Corporation, Melbourne, FL), The results were analyzed according to the gamma evaluation using 3% as the dose difference and 3mm as the distance to the agreement with a 10% threshold. The gamma passing rate should be ≥95%.

### 2.4 Statistical method

Statistical analyses were performed using software package SPSS (version 16.0, IBM Inc.), the results were expressed by $\bar{x}\pm s$. The paired sample t‐Test was used to assess the differences between the two plans. *P* values < 0.05 were considered significant.

## 3 RESULTS

### 3.1 ***The dosimetric comparison of the target volume between the two*** plans

**Figure 4:**
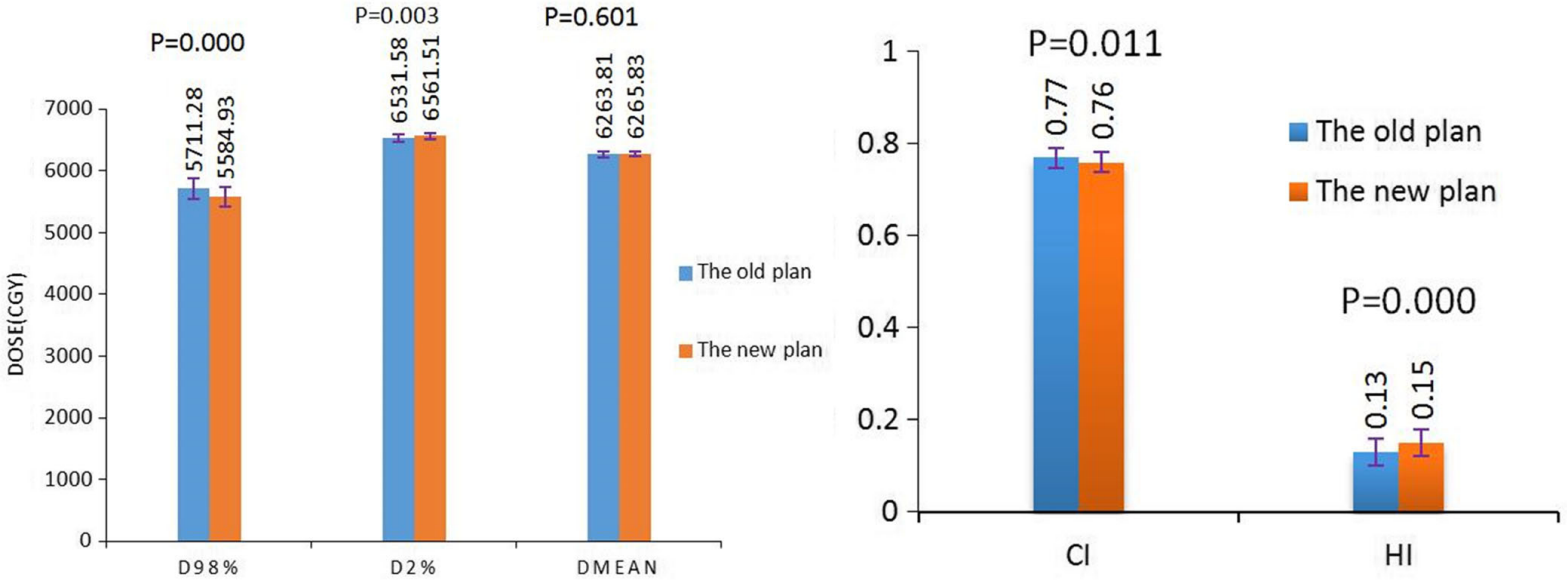
Dosimetric comparison of PTV between the two plans. PTVs. Planning target volumes.

Both the two plans can meet the clinical requirements (the statistical results are presented in Fig. 4). There was negligible difference in the average dose (D_mean_) and approximate maximum dose (D_2%_) of PTV between the two plans. The conformal indices and the homogeneity indices changed little, also negligible. The approximate minimum dose to PTV (D_98%_) reduced by nearly 130 cGy, *P* = 0.000, mainly because of the constrains of the skin dose.

### 3.2 The dosimetric comparison of the OARs between the two plans

**Figure 5:**
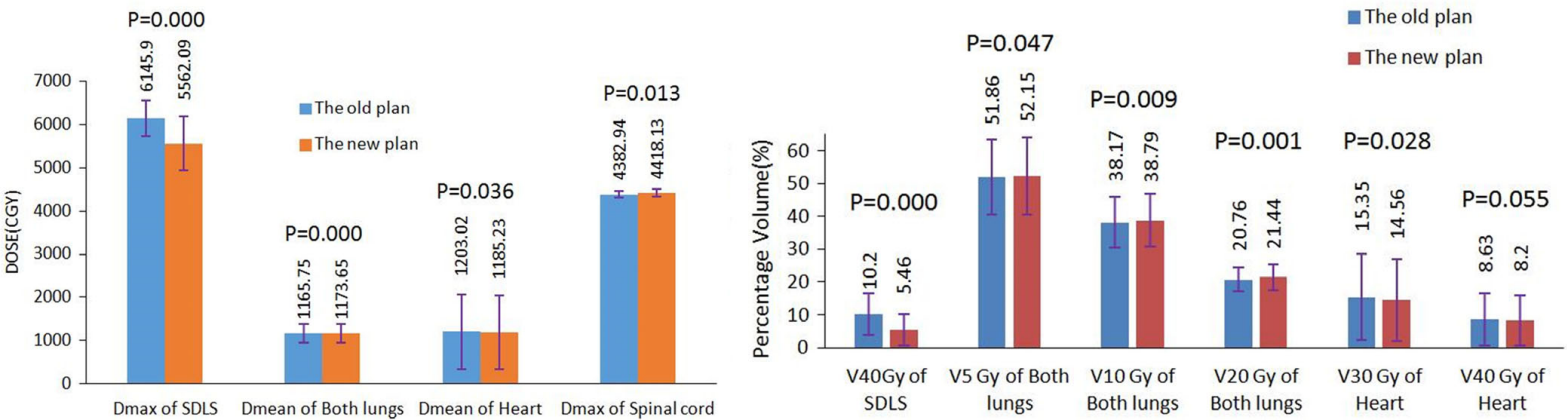
Dosimetric comparisons of the SDLS and OARs between the two plans. SDLS, skin dose limiting structure.

The statistical results of doses to OARs are presented in Fig. 5. The maximum dose of the SDLS of the two plans were 6145.90 ± 416.96 cGy and 5562.09 ± 616.69 cGy, respectively, decreased by nearly 600 cGy, the *P* value was 0; the percentage volume of the SDLS receiving dose of 40 Gy were (10.20 ± 6.36)% and (5.46 ± 4.84)%, respectively, reduced by about 6%, the *P* value was 0, the differences were statistically significant. It is mainly because of the specific dose control of the SDLS after optimization objectives and the objective functions of the SDLS were set before re‐optimization. We can also find out that the dosimetric parameters of both lungs and heart have almost no change after the use of skin dose limiting technique; the maximum dose of the spinal cord increased from 4382.94 ± 72.63 cGy to 4418.13 ± 80.47 cGy, the *P* value was 0.113, statistically insignificant.

## 4 DISCUSSION

Through the comparative study of this paper, we can find that the utilization of this skin dose limiting technique can reduce the dose to skin, while there was little impact on the dose distribution of target volume and the OARs. Figure 4 indicates that the use of skin dose limiting technique has very little impact on the average dose (D_mean_), approximate maximum dose (D_2%_), approximate minimum dose (D_98%_), homogeneity index and conformal index of the target volume. Figure 5 shows that both the maximum dose and V_40Gy_ of the SDLS decreased significantly after the utilization of skin dose limiting technique, while there was nearly no impact on the radiation dose to both lungs and heart. The maximum dose to the spinal cord increased by about 30cGy, mainly because the limitation of the skin dose enlarged the weight of the beam fields penetrated from the spinal cord.

The results of this study were obtained by comparing two treatment plans of 30 patients, we can find a significant skin dose reduction through the use of the skin dose limiting technique demonstrated in this study, this can help us get an ideal skin protection for the patients in the process of radiotherapy, but there was a lack of clinical trial data. Previously, we did not use any techniques specifically aimed at reducing skin dose in treatment planning process. It was only after several times of treatment that the patient experienced severe skin reactions before we revised the treatment plan to obtain a lower skin dose. We will apply the skin dose limiting technique to future clinical work, and observe the symptoms of skin reaction of each patient during the process of radiotherapy.

## 5 CONCLUSION

The purpose of this study was to demonstrate the process of a skin dose limiting technique in radiotherapy treatment planning for esophageal cancer patients. As of now, we have not been able to accurately predict the severity of radiation skin reactions a patient is going to acquire before treatment, but through the comparative study in this paper, we can conclude that the use of the skin dose limiting technique has very little negative effect on the dose distribution of target volume, while it can greatly reduce the radiation dose to the skin, so as to reduce the severity of skin toxicity around the treatment area. The skin dose limiting method demonstrated in this study can be used in other treatment planning techniques such as VMAT, it can also be used in treatment planning for other cancer patients with severe skin reactions during radiotherapy, except for patients with esophageal cancer. In future study, we will continue to carry out comparative study between different treatment planning techniques or different cancer patients whether or not using this skin dose limiting technique.

## CONFLICT OF INTEREST

The authors declare no conflict of interests.

## AUTHORS’ CONTRIBUTIONS

All authors participated in patient treatment planning and plan evaluation, and all authors were involved in the preparation of the manuscript. All authors reviewed and approved the final manuscript.

## ETHICS APPROVAL AND CONSENT TO PARTICIPATE

Not applicable in this section.

## CONSENT FOR PUBLICATION

Not applicable in this section.

## Data Availability

The datasets supporting the conclusions of this article are available from the corresponding author on reasonable request.
